# Anomalous cooling of bosons by dimensional reduction

**DOI:** 10.1126/sciadv.adk6870

**Published:** 2024-02-14

**Authors:** Yanliang Guo, Hepeng Yao, Sudipta Dhar, Lorenzo Pizzino, Milena Horvath, Thierry Giamarchi, Manuele Landini, Hanns-Christoph Nägerl

**Affiliations:** ^1^ Institut für Experimentalphysik und Zentrum für Quantenphysik, Universität Innsbruck, Technikerstraße 25, Innsbruck 6020, Austria.; ^2^ DQMP, University of Geneva, 24 Quai Ernest-Ansermet, Geneva CH-1211, Switzerland.

## Abstract

Cold atomic gases provide a remarkable testbed to study the physics of interacting many-body quantum systems. Temperatures are necessarily nonzero, but cooling to the ultralow temperatures needed for quantum simulation purposes or even simply measuring the temperatures directly on the system can prove to be very challenging tasks. Here, we implement thermometry on strongly interacting two- and one-dimensional Bose gases with high sensitivity in the nanokelvin temperature range. Our method is aided by the fact that the decay of the first-order correlation function is very sensitive to the temperature when interactions are strong. We find that there may be a substantial temperature variation when the three-dimensional quantum gas is cut into two-dimensional slices or into one-dimensional tubes. Notably, the temperature for the one-dimensional case can be much lower than the initial temperature. Our findings show that this decrease results from the interplay of dimensional reduction and strong interactions.

## Teaser

With precise nanokelvin thermometry, anomalous cooling of strongly interacting bosons is observed upon dimensional reduction.

## INTRODUCTION

Cold atomic gases allow a remarkable degree of control over crucial parameters such as the interaction strength and the confining potentials, making them ideal systems for studying the properties of strongly correlated quantum matter (*1*). Their dimensionality can be set freely, via, e.g., optical lattice potentials, and with this, they have enabled the study of a host of properties of interacting one-dimensional (1D) and 2D quantum systems. Highlights in 1D include the observation of bosonic fermionization into the Tonks-Girardeau (TG) state (*2*–*5*), the driving of quench dynamics (*6*–*9*), the investigation into localization effects driven by longitudinal lattices (*10*–*14*) and disorder (*15*–*18*), and the recent observation of spin-charge separation (*19*, *20*). Similarly, 2D systems based on cold atoms have allowed the study of the Berezinskii-Kosterlitz-Thouless transition (*21*, *22*), the investigation of topological properties (*23*, *24*), and the probing of frustrated phases (*25*). In all these works, it has been important to assure that the temperature is low enough compared to the point of quantum degeneracy as nonzero temperatures play a crucial role for the physical phenomena observed.

The presumably lowest temperatures for 2D and 1D systems have so far been achieved by slicing low-entropy 3D samples such as essentially pure atomic Bose-Einstein condensates (BECs) into layers or tubes by means of lattice potentials. While the temperatures of the 3D sources can be determined to high accuracy, with values in the low-nanokelvin range (*1*), estimates of the temperatures of the low-D systems have always been rather vague. Knowing the accurate temperatures for such systems is of broad interest, but determining them is recognized as a difficult task (*16**,* *17**,* *21**,* *26*). Attempts to obtain a temperature value from, e.g., Bragg-spectroscopy data on 1D Luttinger liquids together with exact Bethe-ansatz modeling (*27*) were hampered by rather large systematic uncertainties. Here, we implement precise thermometry at the 1-nK level for strongly interacting 2D and 1D Bose gases. We utilize the fact that the first-order correlation function *g*^(1)^ sensitively depends on temperature when interactions are strong. In the experiment, it is determined from a careful measurement of the momentum distribution, and the results of ab initio state-of-the-art quantum Monte Carlo calculations for various values of the temperature are used as a thermometer scale. We use the thermometer to determine temperatures in 1D that are substantially lower than the starting temperatures, and by this show that dimensional reduction does not necessarily lead to heating, in contrast to previous experimental situations (*17**,* *21**,* *26**,* *28**,* *29*). We are able to interpret this anomalous phenomenon by invoking an entropy argument and find that our data fit well with the theoretical prediction. We attribute our findings to the interplay of tight confinement and strong interactions for bosons that are subject to fermionization.

## RESULTS

**Fig. 1.**
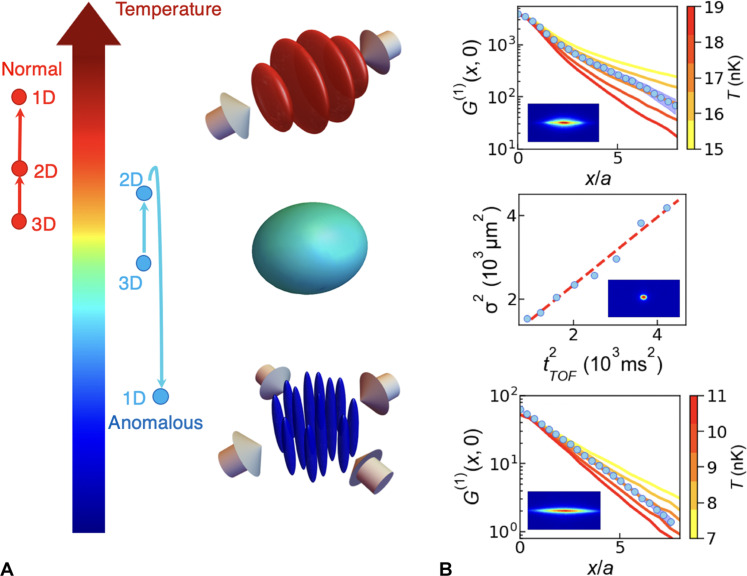
Sketch of the experimental setup and typical thermometry data for the various dimensions. (**A**) The initial nearly spherical 3D BEC (center) is cut either into an ensemble of 2D layers (top) or into an array of 1D tubes (bottom) via the optical force of one or two pairs of counterpropagating and interfering laser beams (arrows). The temperature scale illustrates the normal and anomalous temperature change when the dimensionality is switched for two slightly different initial conditions. (**B**) Example data for the temperature measurements in 2D (top), 3D (middle), and 1D (bottom). For 2D and 1D, the calculated one-body correlation function *G*^(1)^(*x*,0) is plotted as a function of distance *x*/*a* for various temperatures (solid lines, with the temperature indicated by the color coding) and compared to the measured data (blue circles). The experimental statistical error from 20 repetitions is smaller than the size of the symbols. For the 2D case, the system has a weighted atom number ${\bar{N}}_{2D}∼3950$ with radial trapping frequency $\omega_{x}^{2D}/2\pi =10.1\;\text{Hz}$. For the 1D case, the system has a weighted atom number ${\bar{N}}_{1D}∼63$ with longitudinal trapping frequency $\omega_{x}^{1D}/2\pi =14.3\;\text{Hz}$. The scattering length is set to *a*_3*D*_ = 620*a*_0_ for both 2D and 1D. The QMC calculations are under the experimental conditions and its error bars are less than 1%. For the 3D case, a typical TOF dataset with the squared Gaussian waist σ^2^ obtained from a bimodal fit as a function of the squared TOF duration *t*_TOF_ is presented. The linear fit (dashed line) directly gives the 3D temperature. The three insets are example TOF absorption images for the respective dimensionality.

The experiment starts with an interaction-tunable 3D BEC of 1.5 × 10^5^ Cs atoms (*30*) prepared in the lowest magnetic hyperfine state ∣*F*, *m_F_*〉 = ∣ 3,3〉, held in a crossed-beam dipole trap with trap frequencies ω_*x*,*y*,*z*_ = 2π × [18.6(2),19.3(3),26.8(3)] Hz along the three main axes *x*, *y*, and *z* of the setup and levitated along the vertical *x* direction against gravity by a magnetic field gradient. The BEC is in the Thomas-Fermi regime with the 3D s-wave scattering length *a*_3*D*_ tuned to *a*_3*D*_ ≈ 190*a*_0_. One (or two) counterpropagating optical lattice beams along the *z* (and *y*) direction are gradually ramped up in 500 to a potential depth of *V_z_* = 30*E_r_* (*V_y_* = 30*E_r_*), with *E_r_* = π^2^*ℏ*^2^/(2*ma*^2^) the recoil energy, cutting the 3D system into an ensemble of 2D layers that lie in the *x-y* plane (or an array of 1D tubes along the *x*-direction), as sketched in Fig. 1A. Here, *a* = λ/2 is the lattice spacing with λ = 1064.5 nm as the wavelength of the lattice light. After loading the atoms into the lattice, the initial crossed-beam dipole trap is ramped down in 100 ms. For the layers, the trap frequencies are ω_*x*,*y*,*z*_ = 2π × (10.1(2),10.1(2),11*k*) and these change to ω_*x*,*y*,*z*_ = 2π × (14.3(2),11*k*,11*k*) Hz for the set of 1D tubes. The offset magnetic field is then ramped up adiabatically to set *a*_3*D*_ ≈ 620*a*_0_. This takes the 3D BEC into the strictly 2D regime with 2D interaction parameter γ_2*D*_ = 1.5 or into the strictly 1D regime with the Lieb-Linger parameter γ_1*D*_ = 20 (see the Supplementary Materials). For this value, the 1D system is deep in the fermionized TG regime (*3*, *4*).

### Precise nanokelvin thermometry

We now detail the thermometer operation in the various dimensionalities. The method for 3D is standard and has been used widely in the past. In short, the temperature is determined from the expansion rate of the noncondensed fraction of the quantum gas in time-of-flight (TOF) after switching off all trapping fields. Crucially, the interparticle interaction is zeroed at the start of TOF by means of a Feshbach resonance’s zero crossing (*30*) to avoid any residual interaction effects. Bimodal fits on the density profiles for varying TOF times give the 3D temperature (see the Supplementary Materials). Typically, eight different TOF times are chosen, and each time, the experiment is repeated two times. Figure 1B provides the results of a typical measurement, for which we find *T*_3*D*_ = 12.5(4) nK. In 2D and 1D, the interacting gases do not show a bimodal distribution and a Boltzmann fit cannot be done. However, the one-body correlation function $g^{\left(1\right)}\left(x,x\prime ,y,y\prime \right)=\langle {\hat{\Psi }}^{†}\left(x\prime ,y\prime \right)\hat{\Psi }\left(x,y\right)\rangle$ shows a decay that has a strong temperature dependence. In the experiment, we determine it by a measurement of the momentum distribution *n*(*k*) via the TOF technique to obtain the integrated correlation function *G*^(1)^(*x*, *y*) = ∬ *dx*′*dy*′*g*^(1)^(*x*′ + *x*, *y*′ + *y*, *x*′, *y*′) via Fourier transform. We then compare it to the results of an ab initio quantum Monte Carlo (QMC) approach to simulate the system (*31*). Its many-body Hamiltonian is given by

$$\hat{H}=\sum_{j}‍\left[-\frac{\hbar^{2}}{2m}\nabla_{j}^{2}+V\left({\hat{r}}_{j}\right)\right]+\sum_{j<k}‍U\left({\hat{r}}_{j}-{\hat{r}}_{k}\right)$$ (1)

with $U\left(\hat{r}\right)$ being the short-range repulsive two-body interaction and *V*(**r**) being the external harmonic potential. The function *G*^(1)^(*x*, *y*) is then computed for various temperatures using the worm algorithm. Note that simulating one weighted tube (layer) gives us the same result for *G*^(1)^(*x*, *y*) as taking into account the whole atom distribution in the array of tubes (layers) (see the Supplementary Materials). Figure 1B presents typical experimental data for *G*^(1)^(*x*,0) in 1D and 2D and compares the data to the results of the QMC simulations for various temperatures. Clearly, the QMC data serve as a very sensitive ruler for the temperature. For 2D, we obtain *T*_2*D*_ = 17(1) nK, and the temperature in 1D is *T*_1*D*_ = 9(1) nK. The accuracy is set by the systematic discrepancy (see Fig. 1B and fig. S2). Evidently, the system is hotter in 2D, and then colder in 1D. The 1D data can be cross-checked using the analytical form of the correlation function. For a trapped 1D TG gas, it reads *G*^(1)^(*x*) ∼ *e*^−η*x*/*a*^ with η = m *k_B_kTa*/(2*ℏ*^2^*n*_0_) (*32*). With *n*_0_ = 0.9/*a* and η_exp_ = 0.48, we find $T_{1D}^{\text{analytical}}=9.1$ nK. This agrees well with the QMC prediction.

**Fig. 2.**
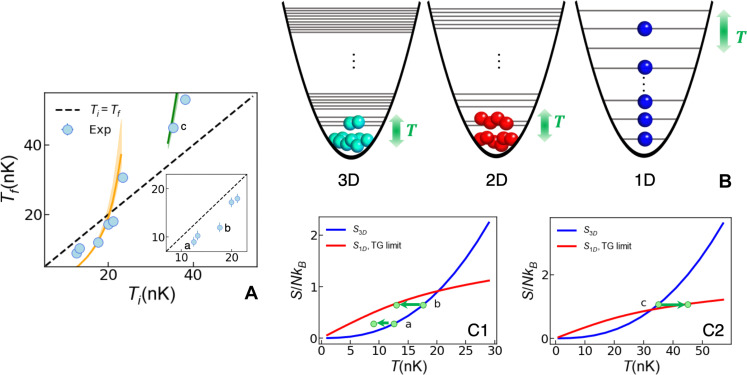
Cooling versus heating and the physical picture behind anomalous cooling. (**A**) The final temperature of the 1D system *T_f_* (blue circles) as a function of the initial temperature of the 3D system *T_i_*. The error bars are smaller than the size of the symbols. The analytical predictions are shown as orange and green solid curves. For these data, the 1D systems are always deeply in the TG regime. The inset displays the low-temperature data for which cooling is observed. The letters a, b, and c mark data points that are referenced in C1 and C2. (**B**) Illustration of the configuration picture in 3D, 2D, and 1D on the basis of the quantum harmonic oscillator. The energy levels indicate the total energy taking all the weakly confined directions into account, with the horizontal axis indicating one of them. The green arrows indicate the effect of the nonzero temperatures. (**C1** and **C2**) The entropy per particle *S*/*Nk_B_* for the 3D trapped system (blue line) and the 1D tubes deeply in the TG regime (red line) as a function of the temperature *T*. The experimental parameters are ω_*x*,*y*,*z*_ = 2π × (18.6,19.3,26.8)Hz and ${\bar{N}}_{1D}=72$ for the data in (C1) and ω_*x*,*y*,*z*_ = 2π × (29.4,27.1,39.9) Hz and ${\bar{N}}_{1D}=120$ for the data in (C2). The green dots reflect the three cases shown in (A), among which a and b show the cooling effect, while c shows heating.

The data above were taken for a specific set of parameters. We now perform cross-checks by varying the initial 3D temperature, the trapping frequency, and the interaction strength to elucidate the mechanism behind the anomalous cooling. For example, by changing the efficiency of the evaporative cooling process in the initial 3D dipole trap, we prepare 3D quantum-degenerate samples at various initial temperatures *T_i_* with varying condensate fractions (see details in the Supplementary Materials). These samples are then transferred into 1D tubes and we measure the final temperature *T_f_* as before. Such temperature data are shown in Fig. 2A. Clearly, anomalous cooling occurs when the initial temperatures are sufficiently low, i.e., 20 nK and below. Typically, we see a decrease of 20 to 40% from the initial 3D temperature, with a temperature difference that is far more than 1-nK thermometer resolution. However, above *T_i_* ≈ 20 nK, the dimensional change leads to heating.

### Anomalous cooling mechanism

Invoking an entropy picture sheds light onto the anomalous cooling phenomenon and demonstrates the important role played by the dimensionality of the quantum many-body system. Figure 2B illustrates the population of the energy levels of the quantum harmonic oscillator in the different dimensionalities. The entropy *S* is known to increase with the number of accessible configurations *W*, i.e., *S* ∼ ln *W*. At high temperatures for an ideal (thermal) gas, it is dominated by the density-of-states effect. The well-known Sackur-Tetrode equation predicts a *d* log *T* dependence of *S*, where *d* = 1,2, or 3 is the integer dimensionality, giving strictly higher *S* in 3D with respect to 1D (assuming ω is fixed). This typically leads to heating when reducing the dimensionality. In the quantum low-temperature regime, the behavior of *S* can change drastically depending on statistical effects. In 3D, the presence of the BEC reduces configurations and leads to a superlinear ∝*T*^5/2^ growth of *S* with *T* (*33*). For 1D gases, fermionization and the absence of condensation lead to sublinear growth. This gives rise to a crossing of the entropy curves, enabling cooling at low enough initial temperatures.

Specifically, when the dimension of the system is reduced from 3D, two processes happen. On one hand, the condensate nature of the initial system is undermined. In 3D, the system is a nearly pure BEC with a small noncondensed fraction at low temperatures. Most of the atoms populate the ground state. In 2D, the nature of the condensate is weakened and the system exists only as a quasicondensate with a decay of the first-order correlations. This suggests an increase in the number of possible configurations *C* in energy space for a given nonzero temperature *T*. In the extreme case of a strongly interacting gas in 1D, the system has fermionized. The particles are filled into the energy levels as ideal fermions and the excitations happen around the Fermi surface. On the other hand, the degeneracy of the energy levels becomes less as the dimensionality is reduced. This leads to the decrease of the number of possible configurations *C*. Thus, as a result of the competition of these two processes, one can reach a situation *C*_1*D*_ > *C*_3*D*_ > *C*_2*D*_. For constant entropy, as a result of careful adiabatic loading the lattice, one may thus obtain *T*_1*D*_ < *T*_3*D*_ < *T*_2*D*_.

This physical picture is confirmed by calculations of the entropy. For 3D trapped bosonic gases in the quantum regime (*33*), the entropy is $S_{3D}=\left(7\mathrm{A\zeta}\left(3\right)/5\sqrt{2}\right){\left(15a_{3D}N/\sigma \right)}^{1/5}{\left(T/\hbar \bar{\omega }\right)}^{5/2}$ with *A* = 10.6, $\sigma =\sqrt{\hbar /m\bar{\omega }}$ is the oscillator length, and $\bar{\omega }={\left(\omega_{x}\omega_{y}\omega_{z}\right)}^{1/3}$ . In the 1D case, the entropy *S*_1*D*_ = −*∂* Ω_TG_/*∂ T* of a TG gas can be computed from the grand potential Ω_TG_ (*34*), with the trap treated under the local density approximation (see calculations in the Supplementary Materials). These entropy curves are shown in Fig. 2C for our set of parameters. Below a certain temperature $T_{i}^{c}$ , the entropy for 1D is higher than in 3D. When keeping the entropy constant, the system’s temperature has to drop when the dimensionality is reduced from 3D to 1D, see, e.g., a and b in Fig. 2A. Our data reflect this. Above $T_{i}^{c}$ , a temperature increase is expected. This is also captured by our data. In Fig. 2A, we add the prediction for the temperature decrease and increase during dimensional reduction. Our data fit the predictions reasonably well. Only for the lowest temperatures do we find a decrease that is not as pronounced as predicted, most likely due to some small nonadiabaticity during the lattice-loading process. We note that the cooling mechanism observed here is reminiscent of the adiabatic demagnetization cooling technique (*35*, *36*). However, in our case, there is no discrete spin degree of freedom into which entropy can be pumped.

### Cooling conditions

**Fig. 3.**
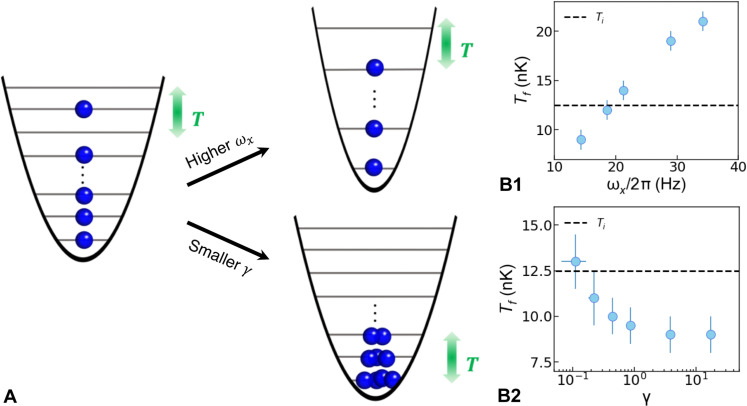
Conditions for anomalous cooling. (**A**) Illustration of the configuration picture for the fermionized TG gas, compared to the one with a higher trapping frequency ω*_x_* or a weaker interaction strength. (**B1** and **B2**) The measured 1D temperature *T_f_* as a function of the longitudinal trapping frequency ω*_x_* (B1) and the interaction strength γ (B2). The black dashed line indicates the initial 3D temperature *T_i_*.

We next turn to the influence of a change of the longitudinal trapping frequency ω*_x_* for the 1D systems. By means of the crossed-beam trap, we can tune ω*_x_*/2π from 14.3(2) Hz to 34.2(3) Hz. Our data, shown in Fig. 3B1, shows that the 1D temperature then varies from 9 to 21 nK. This confirms our entropy and configuration picture: stiffening of the confinement reduces the number of accessible configurations, as shown schematically in Fig. 3A, and hence leads to an increase of the temperature. As is well known, adiabatic compression of a Boltzmann gas leads to heating, and decompression leads to cooling. Our data show that also a TG gas has the same behavior. We note that the lower limit 14.3 Hz for ω*_x_* is set by the residual transversal trapping force of the *y*- and *z*-lattice beams. Reducing this value would require some anti-trapping, which could be done by means of an additional blue-detuned laser beam, and with this even lower 1D temperatures should be possible.

We finally address the role of strong interactions. In the experiment, after preparing the 1D tubes, we ramp *a*_3*D*_ to a value between 7*a*_0_ and 620*a*_0_, with an uncertainty of 3*a*_0_, varying γ between 0.1 and 20 by more than 2 orders of magnitude given our typical atom number $\bar{N}$ . We find a clear temperature dependence on γ as seen in Fig. 3B2. As γ is increased, the 1D temperature drops continuously. Above γ ≈ 1, the temperature settles to a constant value. Evidently, more configurations become accessible as the system starts to fermionize, as sketched in Fig. 3A, leading to a reduction of the temperature, and beyond γ_1*D*_ ≈ 1, the system’s fermionization is complete for a system in equilibrium.

## DISCUSSION

In conclusion, we have realized a thermometer for strongly interacting 1D and 2D quantum gases. We are capable of measuring temperatures for such strongly correlated systems in the low-nanokelvin range with 1-nK precision. With this thermometer, we have found that cooling may occur as the dimensionality is reduced from 3D to 1D, notably different from the heating that has been observed in most experiments so far (*17**,* *21**,* *26**,* *28**,* *29*). We note that recent work (*37*) has also found evidence for cooling in a regime similar to ours, though without further exploration of the mechanism or of the systematics. We have investigated into the requirements of the anomalous cooling effect and have found that extreme conditions of very low initial temperatures, strong interactions, and small 1D trapping frequencies are needed. In view of this effect, one can now optimize the formation process of low-D quantum gases, in particular for the case of box-like trapping conditions (*38*). Next, a variety of phenomena in low-D, for which the temperature plays an important role, can now be explored with much better control, such as Anderson localization (*15*), the pinning (*12*) and Bose glass transitions (*16*, *18*), the dimensional crossover (*39*), and out-of-equilibrium dynamics with, e.g., prethermalization (*9*), dynamical fermionization (*8*), correlated transport (*40*), and the implementation of quantum-field machines (*41*).

## MATERIALS AND METHODS

### Preparation of the experiment

Our experiment starts with a 3D BEC containing around 1.5 × 10^5^ Cs atoms that are prepared in the lowest magnetic hyperfine state ∣*F*, *m_F_*〉 = ∣3,3〉 and confined in a crossed-beam dipole trap with a 3D trapping frequency ω_*x*,*y*,*z*_/2π = (18.6,19.3,26.8) Hz. The atoms are levitated against gravity by means of a magnetic field gradient ∇*B* ∼ 31.1 G/cm oriented along the vertical direction, i.e., *x* direction in the Results section. The 3D s-wave scattering length is set to *a*_3*D*_ ≈ 190*a*_0_, which puts the BEC in the Thomas-Fermi (TF) regime. The details of the trapping and cooling procedures are described in (*30*, *42*). We adiabatically load the BEC into an optical lattice with a lattice depth up to 30*E_r_*, generated from two orthogonally and horizontally propagating retro-reflected laser beams at a wavelength λ = 1064.5 nm, to prepare an ensemble of 2D Bose gases or an array of 1D gases. The lattice results in a tight transversal harmonic trapping frequency of ω*_z_*/2π = 11 kHz. The weak radial confinement in 2D and the longitudinal one in 1D caused by the combined lattice trapping potentials give $\omega_{x}^{2D}/2\pi =10.1\left(2\right)$ Hz and $\omega_{x}^{1D}/2\pi =14.3\left(2\right)$ Hz. During the loading process, almost all layers (2D) or tubes (1D) are in the 2D and 1D TF regime for weak repulsive interactions. We can hence calculate the initial occupation number in each 2D layer or each 1D tube through the global chemical potential and the total atom number (*27*, *43*). When in the lattice, we adiabatically raise the 3D scattering length up to *a*_3*D*_ = 620*a*_0_ by means of a broad magnetic Feshbach resonance (*42*). The ramp time (50 ms) is chosen carefully, i.e., slow enough to avoid any excitations of the breathing modes in the gas.

### The quantum Monte Carlo simulation

The numerical simulations carried out for the thermometry is based on the path-integral Monte Carlo method (*44*). Here, we perform the calculations similar to (*31*, *39*, *45*, *46*). We simulate the system with the Hamiltonian of Eq. 1 in the “Precise nanokelvin thermometry” section with the presence of a harmonic trap, both in the 2D and 1D case. Within the grand canonical ensemble, we simulate the system with a given temperature *T*, interaction strength *g*, and chemical potential μ (equivalently the particle number *N*). Taking advantage of the worm algorithm implementation (*47*, *48*), we run the numerics efficiently and compute the one-body correlation function *G*^(1)^(*x*, *y*) in the open worldline configuration. The numerical calculations make use of the ALPS scheduler library and statistical analysis tools (*49*–*51*).

## Supplementary Materials

### This PDF file includes:

Supplementary Text Figs. S1 to S4 References
