# Supplementary material for: Anomalous cooling of bosons by dimensional reduction
Source: Sci Adv. 2024 Feb 14;10(7):eadk6870. doi: 10.1126/sciadv.adk6870 (PMC10866542; doi:10.1126/sciadv.adk6870)
Supplement: Supplementary file 1 — Supplementary Text Figs. S1 to S4 References [file sciadv.adk6870_sm.pdf]

Science Advances
AAAS

# Supplementary Materials for

## Anomalous cooling of bosons by dimensional reduction

Yanliang Guo *et al.*

Corresponding author: Hanns-Christoph Nägerl, christoph.naegerl@uibk.ac.at

*Sci. Adv.* **10**, eadk6870 (2024)
DOI: 10.1126/sciadv.adk6870

**This PDF file includes:**

Supplementary Text
Figs. S1 to S4
References

# Supplementary Materials

## A. The atom-number distribution

For the calculations of this paper, we use one single layer or one single tube with a weighted average number $\bar{N}$. For 1D, this is given by

$$\bar{N} = \frac{\sum_{i,j} N_{i,j}^2}{\sum_{i,j} N_{i,j}} \tag{S1}$$

with $N_{i,j}$ the atom number of the $i$, $j$-th tube. This standard method has been used in previous works (*27, 29, 43*). It uses the atom number as a weight of itself and it takes into account that tubes with more atoms will contribute more to the physical properties of the system than tubes with less atoms. For 2D, the procedure is similar. The distribution $N_{i,j}$ is computed similarly as in Refs. (*16, 17, 27, 52*). Since the lattice is loaded adiabatically and the interactions are controlled to be small during the loading process, we can define a global chemical potential and assume the local chemical potential of each tube $\mu_{i,j}$ follows a Thomas-Fermi distribution, namely $\mu_{i,j} = \mu - 1/2ma^2(\omega_x^2 i^2 + \omega_y^2 j^2)$. Then, one can assume that the particles are redistributed between the tubes during the ramping-up process of the lattices. By conserving the total particle number $N = \sum_{i,j} N_{i,j}(\mu)$, one can numerically compute the final tube distribution $N_{i,j}$. In the following, we check that this method is proper for the physical quantities studied here. First, we compute the correlation function $G^{(1)}(x, 0)$ for the 1D systems as done for the QMC calculations shown in Fig. 1B of the main text, see Fig. S1 A. The results for one single weighted tube agree with the calculations for the full tube distribution. The difference is less than $5\%$, showing that the use of the weighted average atom number is good enough for estimating the temperature in low dimensions. Second, we check that also the result for the entropy using one weighted tube agrees with the calculation using the distribution of tubes. The results are shown Fig. S1 B for a TG gas under the condition $\bar{N} = 40$ and $\omega_x = 2\pi \times 14.3$ Hz. Also here we find good agreement.

The interaction strengths in 1D and 2D are then determined by the using the weighted atom number. In 1D, the coupling constant is (*1, 53*)

$$g_{\rm 1D} = \frac{2\hbar^2 a_{\rm 3D}}{m l_\perp^2}\left(1 - \frac{1.036 a_{\rm 3D}}{l_\perp}\right)^{-1} \tag{S2}$$

with $l_\perp = \sqrt{\hbar/m\omega_\perp}$ the characteristic transverse length, and the 1D Lieb-Liniger parameter is hence given by $\gamma_{\rm 1D} = m g_{\rm 1D}/\hbar^2\, \bar{n} a$ with the density $\bar{n}$ of the weighted average tube. In 2D, the coupling constant writes (*1, 54*)

$$g_{\rm 2D} \simeq \frac{2\hbar^2\sqrt{2\pi}}{m l_\perp/a_{\rm 3D} + 1/\sqrt{2\pi}\,\ln(1/\pi q^2 l_\perp^2)} \tag{S3}$$

with $q = \sqrt{2m|\mu|/\hbar^2}$ the quasi-momentum, and $\mu$ the chemical potential corresponding to the weighted average layer. The 2D interaction parameter thus is $\gamma_{\rm 2D} = m g_{\rm 2D}/\hbar^2$.

## B. Thermometer in the weakly-interacting regime

For most of the cases in the main text, we estimate the temperature of the system by using the one-body correlation function $G^{(1)}(x, 0)$. In the strongly interacting regime, this quantity follows a purely exponential decay and its decay exponent has a strong and clear dependence on temperature. However, in the weakly interacting regime, the correlation decay becomes slower and it is not a purely exponential decay, which makes the analysis more complicated. In this case, we extract the temperature of the system directly from the momentum distribution $n(k_x)$. This method has been used to estimate the temperature of 1D weakly-interacting gases in previous experimental studies (*26, 55, 56*). In Fig. S2, we present one example for $\underline{N}_{\rm 1D} \sim 70$, longitudinal trapping frequency $\omega_x/2\pi = 14.3$ Hz and 1D coupling constant $m g_{\rm 1D} a/\hbar^2 = 0.11$, resulting in $\gamma \sim 0.2$. Our QMC simulations provide a thermometer scale with a temperature sensitivity of $\delta T \approx 1.5$ nK, see the colored solid curves. With the data from the range $k a_{\rm 1D} < 20$, the measurement suggests 1D temperature $T_{\rm 1D} = 11\,{\rm nK} \pm 1.5$ nK. Notably, such an estimate for weakly-interacting gases according to the small-$k$ part of the momentum distribution is

similar to the one mentioned in Refs. (*26, 55*). Instead of using the analytical formula for the width of the distribution, our calculation directly simulates the continuous trapped system and avoids the local density approximation. We note that the resolution of the thermometer in the regime of weak interactions is not quite as good as the resolution the regime of strong interactions. This is due to the fact that for weak interactions the system is less sensitive to the temperature due to the more pronounced quantum coherence.

## C. The preparation of different initial 3D temperatures

In Fig. 2 A of the main text we show how the final temperature of the 1D system varies with the initial 3D temperature. In the experiment, we prepare 3D gases at different temperatures by changing the efficiency of the evaporative cooling process, i.e., varying the trap depth through altering the power of the crossed dipole beams. However, once the trap depth is modified, the 3D trapping frequency and the weighted atom number in 1D are changed, resulting in different entropy for both cases in 3D and 1D, as shown in Fig 2 C. To systematically compare the measured 1D temperature to the theoretical predicted value that drawn as the orange and the green solid lines in Fig 2A , we have to keep the 3D trapping frequency constant. So, after evaporative cooling, we first ramp up the power of the crossed dipole beams to a fixed value, which provides a constant 3D trapping frequency $\omega_{\rm 3D} = 2\pi \times (18.6, 19.3, 26.8)\rm Hz$ and then load atoms into the lattice. However, such a trap depth limits the reachable highest temperatures for a 3D gas. For preparing a hotter 3D cloud, we need to increase the power of the crossed-dipole beams setting the final trapping frequency to $\omega_{\rm 3D} = 2\pi \times (29.4, 27.1, 39.9)\rm Hz$. This allows us to measure the 1D temperatures to the predicted values given by the entropy curves in Fig 2 C1 and C2.

Finally, we argue that the process of measuring the 3D temperatures is well-defined. The 3D temperatures discussed in the main text are all measured after ramping up the crossed dipole-

trap beams to obtain the desired $\omega_{3D}$. In addition, the inter-particle interaction is switched off during TOF expansion. The resulting true momentum distribution $n(k_x)$ shows an obvious non-condensed part, and hence it can be perfectly fitted by a bimodal (Gaussian plus Thomas-Fermi) distribution, see Fig S3, allowing us to extract the 3D temperature accurately as shown in Fig. 1B.

## D. The imaging setup

Our setup consists of two lattice beams perpendicular to each other along the $y$- and $z$-axes. There is an angle of $\theta \sim 57^\circ$ between the propagation axis of the imaging beam and the $y$-direction, see Fig. S4. The $x$-axis is perpendicular to the imaging beam. To determine the temperature in any dimension, we measure the momentum distributions $n(k_x)$ and hence the correlation functions $G^{(1)}(x,0)$ along the longitudinal direction $x$. They are thus not affected by the imaging angle.

## E. The entropy of the TG gas

The entropy of the TG gas in an harmonic trap can be computed based on the equations in Ref. (*34*). The grand potential density $\Omega$ of a homogeneous TG system can be expressed to the lowest order as

$$\Omega(\mu,T) = -\frac{\sqrt{m}(k_{\mathrm{B}}T)^{3/2}}{\hbar\sqrt{2\pi}} f_{3/2}(\mu/k_{\mathrm{B}}T) \tag{S4}$$

with $\mu$ the chemical potential and $f_\nu(x)$ the completed Fermi-Dirac integral at index $j$. Correspondingly, the entropy density $s$ of this point can be obtained by

$$s(\mu,T) = -\frac{\partial\Omega(\mu,T)}{\partial T}\bigg|_{\mu}. \tag{S5}$$

Then, the presence of the harmonic trap can be taken care of by using the local density approximation (LDA) by taking the equation of state for the low temperature TG gas, namely

$$n = \sqrt{\frac{2m\mu}{\pi^2\hbar^2}}. \tag{S6}$$

This means that the entropy of the trapped system can be expressed as

$$S_{\text{trap}}(N,T) = \int_{-R}^{+R} dx\ s(\mu(x),T) \tag{S7}$$

under the constrain of the total number of particles $\int_{-R}^{+R} dx\ n(\mu - 1/2m\omega^2x^2) = N$.

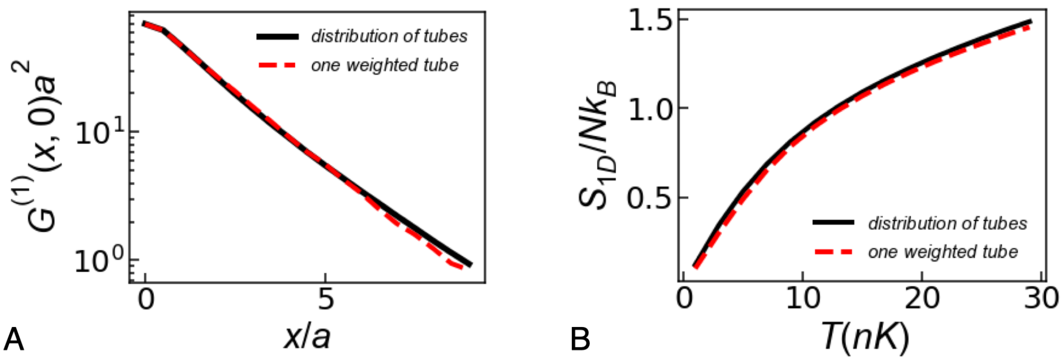


Figure S1: **Check that $\overline{N}$ is a proper input for the numerical calculations.** (A) The correlation function $G^{(1)}(x, 0)$ as a function of the position $x/a$ for the same parameters as for the 1D experimental data shown in Fig. 1B of the main text and for $T = 9$ nK. (B) The entropy per particle $S/Nk_\text{B}$ as a function of temperature $T$ for a TG gas with $\bar{N} = 40$ and $\omega_x = 2\pi \times 14.3$ Hz. The red dashed line indicates the calculation for the case of one weighted tube, while the black solid line assumes the distribution of tubes.

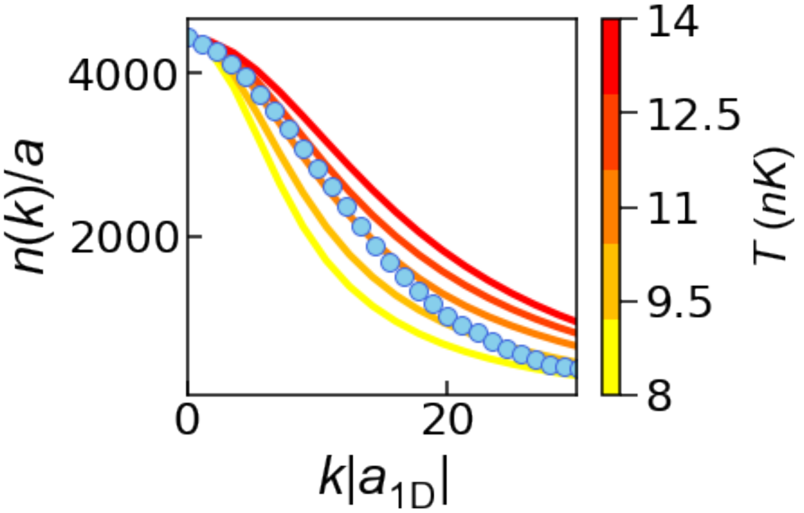


Figure S2: **Thermometer for 1D weakly-interacting systems based on the momentum distribution.** The blue balls are experimental data with weighted atom number $\overline{N}_\text{1D} \sim 70$, longitudinal trapping frequency $\omega_x/2\pi = 14.3$ Hz and 1D coupling constant $mg_\text{1D}a/\hbar^2 = 0.11$. The momentum axis is rescaled by $a_\text{1D} = -2\hbar^2/mg_\text{1D}$. The solid lines are the results of QMC simulations for the same conditions as the experiment for various values of the temperature as indicated.

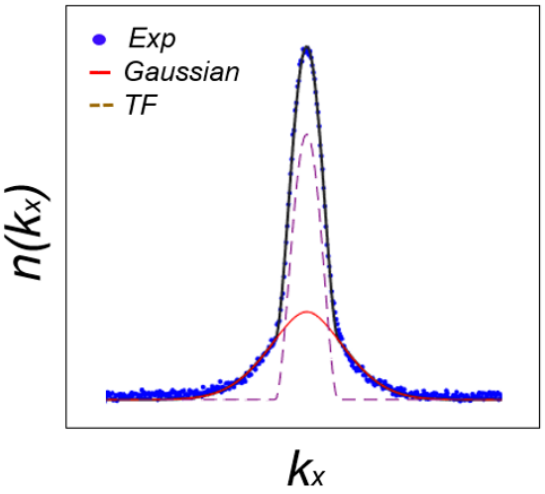


Figure S3: **Bimodal fit for the momentum distribution** $n(k_x)$ **in 3D.** Example of $n(k_x)$ for the 3D gas with $50$-ms TOF for the data shown in Fig. 1 (B) in the main text.

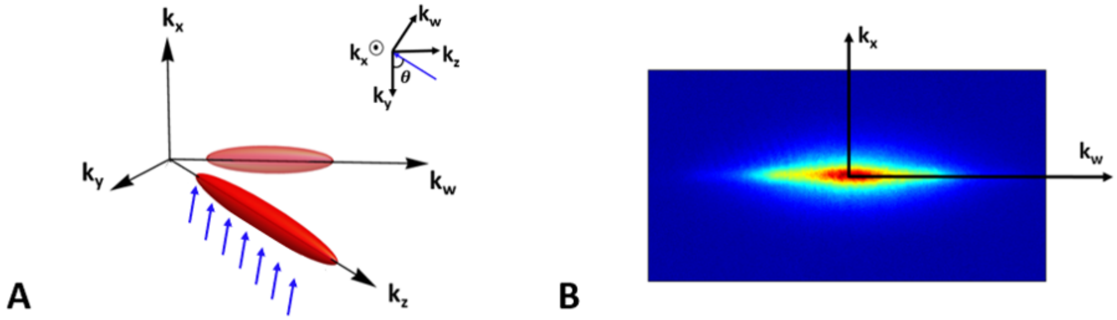


Figure S4: **A schematic of our imaging setup and an example of an image.** (A) The vectors $k_y$, $k_z$, $k_w$ and the imaging direction (blue arrows) all lie in one plane, with $k_x$ perpendicular to this plane, see also the inset, which shows all vectors after a rotation. The red 3D ellipsoid along the $k_z$-direction indicates the atomic cloud after TOF starting from an ensemble of 2D layers for $V_y = 0E_r$. The light red 2D ellipsoid along the $k_w$-direction is the shadow in our absorption image. (B) The projected image after TOF for the 2D case.

**REFERENCES AND NOTES**

1. I. Bloch, J. Dalibard, W. Zwerger, Many-body physics with ultracold gases, *Rev. Mod. Phys.* **80**, 885–964 (2008).

2. B. Paredes, A. Widera, V. Murg, O. Mandel, S. Fölling, I. Cirac, G. V. Shlyapnikov, T. W. Hänsch, I. Bloch, Tonks-Girardeau gas of ultracold atoms in an optical lattice *Nature* **429**, 277–281 (2004).

3. T. Kinoshita, T. Wenger, D. S. Weiss, Observation of a one-dimensional Tonks-Girardeau gas, *Science* **305**, 1125–1128 (2004).

4. E. Haller, M. Gustavsson, M. J. Mark, J. G. Danzl, R. Hart, G. Pupillo, H.-C. Nägerl, Realization of an excited, strongly-correlated quantum gas phase *Science* **325**, 1224–1227 (2009).

5. F. Meinert, M. Knap, E. Kirilov, K. Jag-Lauber, M. B. Zvonarev, E. Demler, H.-C. Nägerl, Bloch oscillations in the absence of a lattice *Science* **356**, 945–948 (2017).

6. S. Hofferberth, I. Lesanovsky, B. Fischer, T. Schumm, J. Schmiedmayer, Non-equilibrium coherence dynamics in one-dimensional Bose gases, *Nature* **449**, 324–327 (2007).

7. F. Meinert, M. J. Mark, E. Kirilov, K. Lauber, P. Weinmann, M. Gröbner, A. J. Daley, H.-C. Nägerl, Observation of many-body dynamics in long-range tunneling after a quantum quench *Science* **344**, 1259–1262 (2014).

8. J. Wilson, N. Malvania, Y. Le, Y. Zhang, M. Rigol, D. S. Weiss, Observation of dynamical fermionization *Science* **367**, 1461–1464 (2020).

9. Y. Le, Y. Zhang, S. Gopalakrishnan, M. Rigol, D. S. Weiss, Observation of hydrodynamization and local prethermalization in 1D Bose gases *Nature* **618**, 494–499 (2023).

10. T. Stöferle, H. Moritz, C. Schori, M. Köhl, T. Esslinger, Transition from a strongly interacting 1D superfluid to a mott insulator *Phys. Rev. Lett.* **92**, 130403 (2004).

11. D. Clément, N. Fabbri, L. Fallani, C. Fort, M. Inguscio, Exploring correlated 1D Bose gases from the superfluid to the Mott-insulator state by inelastic light scattering *Phys. Rev. Lett.* **102**, 155301 (2009).

12. E. Haller, R. Hart, M. J. Mark, J. G. Danzl, L. Reichsöllner, M. Gustavsson, M. Dalmonte, G. Pupillo, H.-C. Nägerl, Pinning quantum phase transition for a Luttinger liquid of strongly interacting bosons *Nature* **466**, 597–600 (2010).

13. N. Fabbri, S. D. Huber, D. Clément, L. Fallani, C. Fort, M. Inguscio, E. Altman, Quasiparticle dynamics in a Bose insulator probed by interband Bragg spectroscopy *Phys. Rev. Lett.* **109**, 055301 (2012).

14. G. Boéris, L. Gori, M. D. Hoogerland, A. Kumar, E. Lucioni, L. Tanzi, M. Inguscio, T. Giamarchi, C. D'Errico, G. Carleo, G. Modugno, L. S. Palencia, Mott transition for strongly interacting one-dimensional bosons in a shallow periodic potential *Phys. Rev. A* **93**, 011601 (2016).

15. J. Billy, V. Josse, Z. Zuo, A. Bernard, B. Hambrecht, P. Lugan, D. Clément, L. Sanchez-Palencia, P. Bouyer, A. Aspect, Direct observation of Anderson localization of matter waves in a controlled disorder *Nature* **453**, 891–894 (2008).

16. C. D'Errico, E. Lucioni, L. Tanzi, L. Gori, G. Roux, I. P. McCulloch, T. Giamarchi, M. Inguscio, G. Modugno, Observation of a disordered bosonic insulator from weak to strong interactions *Phys. Rev. Lett.* **113**, 095301 (2014).

17. L. Gori, T. Barthel, A. Kumar, E. Lucioni, L. Tanzi, M. Inguscio, G. Modugno, T. Giamarchi, C. D'Errico, G. Roux, Finite-temperature effects on interacting bosonic one-dimensional systems in disordered lattices *Phys. Rev. A* **93**, 033650 (2016).

18. M. Sbroscia, K. Viebahn, E. Carter, J.-C. Yu, A. Gaunt, U. Schneider, Observing localization in a 2D quasicrystalline optical lattice *Phys. Rev. Lett.* **125**, 200604 (2020).

19. J. Vijayan, P. Sompet, G. Salomon, J. Koepsell, S. Hirthe, A. Bohrdt, F. Grusdt, I. Bloch, C. Gross, Time-resolved observation of spin-charge deconfinement in fermionic Hubbard chains *Science* **367**, 186–189 (2020).

20. R. Senaratne, D. Cavazos-Cavazos, S. Wang, F. He, Y.-T. Chang, A. Kafle, H. Pu, X.-W. Guan, R. G. Hulet, Spin-charge separation in a one-dimensional Fermi gas with tunable interactions *Science* **376**, 1305–1308 (2022).

21. Z. Hadzibabic, P. Krüger, M. Cheneau, B. Battelier, J. Dalibard, Berezinskii-Kosterlitz-Thouless crossover in a trapped atomic gas, *Nature* **441**, 1118–1121 (2006).

22. L.-C. Ha, C.-L. Hung, X. Zhang, U. Eismann, S.-K. Tung, C. Chin, Strongly interacting two-dimensional Bose gases *Phys. Rev. Lett.* **110**, 145302 (2013).

23. N. Goldman, J. Dalibard, A. Dauphin, F. Gerbier, M. Lewenstein, P. Zoller, I. B. Spielman, Direct imaging of topological edge states in cold-atom systems *Proc. Natl. Acad. Sci.* **110**, 6736–6741 (2013).

24. M. Tarnowski, F. N. Ünal, N. Fläschner, B. S. Rem, A. Eckardt, K. Sengstock, C. Weitenberg, Measuring topology from dynamics by obtaining the Chern number from a linking number *Nat. Commun.* **10**, 1728 (2019).

25. J. Struck, C. Ölschläger, R. Le Targat, P. Soltan-Panahi, A. Eckardt, M. Lewenstein, P. Windpassinger, K. Sengstock Quantum simulation of frustrated classical magnetism in triangular optical lattices *Science* **333**, 996–999 (2011).

26. N. Fabbri, D. Clément, L. Fallani, C. Fort, M. Inguscio, Momentum-resolved study of an array of one-dimensional strongly phase-fluctuating Bose gases *Phys. Rev. A* **83**, 031604 (2011).

27. F. Meinert, M. Panfil, M. J. Mark, K. Lauber, J.-S. Caux, H.-C. Nägerl, Probing the excitations of a Lieb-Liniger gas from weak to strong coupling *Phys. Rev. Lett.* **115**, 085301 (2015).

28. N. Fabbri, M. Panfil, D. Clément, L. Fallani, M. Inguscio, C. Fort, J.-S. Caux, Dynamical structure factor of one-dimensional Bose gases: Experimental signatures of beyond-Luttinger-liquid physics *Phys. Rev. A* **91**, 043617 (2015).

29. C. Li, T. Zhou, I. Mazets, H.-P. Stimming, F. S. Møller, Z. Zhu, Y. Zhai, W. Xiong, X. Zhou, X. Chen, J. Schmiedmayer, Relaxation of bosons in one dimension and the onset of dimensional crossover *Sci Post Phys.* **9**, 058 (2020).

30. T. Kraemer, J. Herbig, M. Mark, T. Weber, C. Chin, H.-C. Nägerl, R. Grimm, Optimized production of a cesium Bose-Einstein condensate *Appl. Phys. B* **79**, 1013–1019 (2004).

31. H. Yao, L. Pizzino, T. Giamarchi, Strongly-interacting bosons at 2D-1D dimensional crossover, *SciPost Phys.* **15**, 050 (2023).

32. A. Minguzzi, P. Vignolo, Strongly interacting trapped one-dimensional quantum gases: Exact solution *AVS Quantum Science* **4**, doi.org/10.1116/5.0077423 (2022).

33. T.-L. Ho, Q. Zhou, Intrinsic heating and cooling in adiabatic processes for bosons in optical lattices *Phys. Rev. Lett.* **99**, 120404 (2007).

34. W. Xu, M. Rigol, Universal scaling of density and momentum distributions in Lieb-Liniger gases *Phys. Rev. A* **92**, 063623 (2015).

35. W. F. Giauque, D. P. MacDougall, Attainment of temperatures below 1° absolute by demagnetization of Gd2(SO4)3.8H2O *Phys. Rev.* **43**, 768 (1933).

36. M. Fattori, T. Koch, S. Goetz, A. Griesmaier, S. Hensler, J. Stuhler, T. Pfau, Demagnetization cooling of a gas *Nat. Phys.*, 765–768 (2006).

37. K.-Y. Li, Y. Zhang, K. Yang, K.-Y. Lin, S. Gopalakrishnan, M. Rigol, B. L. Lev, Rapidity and momentum distributions of one-dimensional dipolar quantum gases *Phys. Rev. A* **107**, L061302 (2023).

38. N. Navon, R. P. Smith, and Z. Hadzibabic, Quantum gases in optical boxes, *Nat. Phys.* **17**, 1334–1341 (2021).

39. Y. Guo, H. Yao, S. Ramanjanappa, S. Dhar, M. Horvath, L. Pizzino, T. Giamarchi, M. Landini, H.-C. Nägerl, Experimental observation of the 2d-1d dimensional crossover in strongly interacting ultracold bosons, arXiv:2308.00411 [quant-ph] (2023).

40. C. J. M. Mathy, M. B. Zvonarev, E. Demler, Quantum flutter of supersonic particles in one-dimensional quantum liquids *Nat. Phys.* **8**, 881–886 (2012).

41. M. Gluza, J. Sabino, N. H. Y. Ng, G. Vitagliano, M. Pezzutto, Y. Omar, I. Mazets, M. Huber, J. Schmiedmayer, J. Eisert, Quantum field thermal machines *PRX Quantum.* **2**, 030310 (2021).

42. V. Weber, J. Herbig, M. Mark, H.-C. Nägerl, R. Grimm, Bose-Einstein condensation of cesium *Science* **299**, 232–235 (2003).

43. E. Haller, M. Rabie, M. J. Mark, J. G. Danzl, R. Hart, K. Lauber, G. Pupillo, H.-C. Nägerl, Three-body correlation functions and recombination rates for bosons in three dimensions and one dimension *Phys. Rev. Lett.* **107**, 230404 (2011).

44. D. M. Ceperley, Path integrals in the theory of condensed helium *Rev. Mod. Phys.* **67**, 279–355 (1995).

45. H. Yao, D. Clément, A. Minguzzi, P. Vignolo, L. Sanchez-Palencia, Tan's contact for trapped Lieb-Liniger bosons at finite temperature, *Phys. Rev. Lett.* **121**, 220402 (2018).

46. H. Yao, T. Giamarchi, L. Sanchez-Palencia, Lieb-Liniger bosons in a shallow quasiperiodic potential: Bose glass phase and fractal Mott lobes *Phys. Rev. Lett.* **125**, 060401 (2020).

47. M. Boninsegni, Nikolay Prokof'ev, B. Svistunov, Worm algorithm for continuous-space path integral Monte Carlo simulations *Phys. Rev. Lett.* **96**, 070601 (2006).

48. M. Boninsegni, N. V. Prokof'ev, B. V. Svistunov, Worm algorithm and diagrammatic Monte Carlo: A new approach to continuous-space path integral Monte Carlo simulations *Phys. Rev. E* **74**, 036701 (2006).

49. M. Troyer, B. Ammon, E. Heeb, in *International Symposium on Computing in Object-Oriented Parallel Environments* (Springer, 1998), pp. 191–198.

50. A.F. Albuquerque, F. Alet, P. Corboz, P. Dayal, A. Feiguin, S. Fuchs, L. Gamper, E. Gull, S. Gürtler, A. Honecker, R. Igarashi, M. Körner, A. Kozhevnikov, A. Läuchli, S.R. Manmana, M. Matsumoto, I.P. Mc Culloch, F. Michel, R.M. Noack, G. Pawłowski, L. Pollet, T. Pruschke, U. Schollwöck, S. Todo, S. Trebst, M. Troyer, P. Werner, S. Wessel; ALPS collaboration, The ALPS project release 1.3: Open-source software for strongly correlated systems *J. Magn. Magn. Mater.* **310**, 1187–1193 (2007).

51. B. Bauer, L D Carr, H G Evertz, A. Feiguin, J. Freire, S. Fuchs, L. Gamper, J. Gukelberger, E. Gull, S. Guertler, A. Hehn, R. Igarashi, S V Isakov, D. Koop, P N Ma, P. Mates, H. Matsuo, O. Parcollet, G. Pawłowski, J D Picon, L. Pollet, E. Santos, V W Scarola, U. Schollwöck, C. Silva, B. Surer, S. Todo, S. Trebst, M. Troyer, M L Wall, P. Werner, S. Wessel, The ALPS project release 2.0: Open source software for strongly correlated systems, *J. Stat. Mech. Theory Exp.* **05**, P05001 (2011).

52. M. Landini, N. Dogra, K. Kroeger, L. Hruby, T. Donner, T. Esslinger Formation of a spin texture in a quantum gas coupled to a cavity *Phys. Rev. Lett.* **120**, 223602 (2018).

53. M. A. Cazalilla, R. Citro, T. Giamarchi, E. Orignac, M. Rigol, One dimensional bosons: From condensed matter systems to ultracold gases *Rev. Mod. Phys.* **83**, 1405–1466 (2011).

54. Z. Hadzibabic, J. Dalibard, Two-dimensional Bose fluids: An atomic physics perspective *Riv. del Nuovo Cim.* **34**, 389 (2011).

55. F. Gerbier, J. H. Thywissen, S. Richard, M. Hugbart, P. Bouyer, A. Aspect, Momentum distribution and correlation function of quasicondensates in elongated traps, *Phys. Rev. A* **67**, 051602 (2003).

56. B. Yang, Y.-Y. Chen, Y.-G. Zheng, H. Sun, H.-N. Dai, X.-W. Guan, Z.-S. Yuan, J.-W. Pan, Quantum criticality and the Tomonaga-Luttinger liquid in one-dimensional bose gases *Phys. Rev. Lett.* **119**, 165701 (2017).
